# Sustainable Antimicrobial Textiles Functionalized with Gold and Silver Nanoparticles Biosynthesized Using *Undaria pinnatifida* Extracts

**DOI:** 10.3390/nano16171112

**Published:** 2026-09-03

**Authors:** João Abreu, Mário Fernandes, Ana Rita Bragança, Bruna Silva, Diana Rocha, Raúl Machado, Artur Ribeiro, Maria Carmen Rodríguez-Argüelles, Carla Silva, Andreia C. Gomes

**Affiliations:** 1 Centre of Molecular and Environmental Biology (CBMA)/Aquatic Research Network (ARNET) Associate Laboratory, Universidade do Minho, Campus de Gualtar, 4710-057 Braga, Portugal; joaopaulogavetaabreu@gmail.com (J.A.); mariofernandesvsc@gmail.com (M.F.); id11146@alunos.uminho.pt (A.R.B.); id11148@uminho.pt (B.S.); raulmachado@bio.uminho.pt (R.M.); 2 Institute of Science and Innovation for Sustainability (IB-S), Universidade do Minho, Campus de Gualtar, 4710-057 Braga, Portugal; 3 Centre of Biological Engineering, University of Minho, Campus de Gualtar, 4710-057 Braga, Portugal; diana.rochaa97@hotmail.com (D.R.); arturibeiro@ceb.uminho.pt (A.R.); 4 LABBELS—Associate Laboratory, 4710-057 Braga, Portugal; 5 Departamento de Química Inorgánica, Universidade de Vigo, 36310 Vigo, Spain; mcarmen@uvigo.es

**Keywords:** *Undaria pinnatifida*, green synthesis, gold nanoparticles, silver nanoparticles, antimicrobial activity, functionalized textiles

## Abstract

Functionalizing textiles with nanoparticles is a promising strategy for developing sustainable materials with antimicrobial activity and reduced potential for resistance. This study aimed to develop antimicrobial cotton and polyester (PE) textiles functionalized with gold and silver nanoparticles (Au@UP and Ag@UP) biosynthesized using *Undaria pinnatifida* (UP) aqueous extracts. The nanoparticle-functionalized textiles were characterized by Ultraviolet–visible spectroscopy (UV-vis), scanning electron microscopy (SEM), and fourier transform infrared spectroscopy (FTIR), and nanoparticle attachment–detachment and stability were evaluated on both substrates. Antioxidant activity was assessed by the 2,2-diphenyl-1-picrylhydrazyl (DPPH) assay, while antimicrobial activity against *Staphylococcus aureus* and *Pseudomonas aeruginosa* and cytocompatibility using the L-929 cell line were determined. UV–vis spectroscopy revealed residual release, while SEM, UV–vis, and FTIR confirmed nanoparticle binding. Cotton exhibited higher nanoparticle affinity and stability (KS = 0.5007 for Au@UP and 0.4817 for Ag@UP), attributed to its abundance of reactive hydroxyl groups. Although antioxidant activity decreased after nanoparticle binding (<5%), functionalized cotton gauzes retained antimicrobial activity. Au@UP and Ag@UP textiles were non-cytotoxic (>80% cell viability) and inhibited the growth of *S. aureus* (~15% and 90%, respectively) and *P. aeruginosa* (~90% for both). These findings support the potential of UP-mediated nanoparticle-functionalized textiles, particularly Ag@UP-containing materials, as sustainable antimicrobial surfaces.

## 1. Introduction

Healthcare-associated infections remain a major global challenge, particularly due to the increasing prevalence of multidrug-resistant bacteria and the limited efficacy of conventional antimicrobial treatments [1]. In this context, antimicrobial textiles have emerged as promising materials for reducing microbial transmission and preventing infections in medical and healthcare settings [2]. Among the various strategies explored, textile functionalization with bioactive agents has attracted considerable attention because it enables the development of materials with enhanced antimicrobial performance while maintaining desirable properties such as biocompatibility, durability and biodegradability [3,4]. In particular, the use of naturally derived functionalizing agents represents an attractive approach for developing safer and more sustainable antimicrobial textile systems [5]. The choice of textile substrate is also an important consideration, as the physicochemical properties of the material can influence both its suitability for biomedical applications and the incorporation and performance of antimicrobial agents.

Cotton is a cellulosic material widely used in the biomedical industry due to its favorable mechanical properties, including absorbency, permeability and biodegradability [6]. Polyester-based textiles also exhibit excellent characteristics for medical applications, particularly due to their inert nature and generally high biocompatibility [3,7]. As both of these types of textiles are biocompatible and can be functionalized with non-toxic agents to impart additional bioactivities, such as antimicrobial properties, they have been widely used [3,4,6]. Thus, cotton- and polyester-based materials provide relevant and distinct textile substrates for investigating the incorporation of antimicrobial agents and their resulting biological performance.

Among the different approaches used to impart antimicrobial properties to such substrates, metal-based nanoparticles (NPs) have received considerable attention. Metal-based nanoparticles (NPs) have emerged as promising antimicrobial agents due to their broad-spectrum activity and low likelihood of inducing microbial resistance [3]. These may include zinc, copper, silver, gold, titanium oxide and other metallic nanoparticles [8,9,10,11]. Their antimicrobial potential arises from direct interactions with the bacterial cell wall and membranes and includes mechanisms such as oxidative stress induction, metal ion release and non-oxidative mechanisms [12]. Since these structural barriers are highly conserved, only extensive genetic mutation would alter bacterial susceptibility to NPs, making the development of resistance unlikely [13]. In particular, silver and gold NPs have attracted considerable interest because they can interact directly with bacterial cell structures and disrupt essential cellular processes without requiring specific molecular targets [14,15,16,17]. Silver nanoparticles are widely recognized for their potent antibacterial activity and chemical stability, whereas gold nanoparticles offer additional advantages such as high biocompatibility and unique physicochemical properties [18,19]. Therefore, the incorporation of silver and gold NPs into textile substrates represents a potentially effective strategy for combining the functional properties of textiles with the antimicrobial activity of nanomaterials.

Beyond the choice of nanoparticle, the method used for their synthesis is also relevant to the development of antimicrobial textile systems, particularly when sustainability and biocompatibility are considered. Green synthesis has emerged as a sustainable, low-cost and non-toxic approach for NP production [20]. This strategy combines the intrinsic properties of NPs with bioactive compounds from organisms, such as marine seaweeds, that often possess potent defense mechanisms against ecological pressures [21,22]. Marine macroalgae such as *Undaria pinnatifida* (UP) contain polysaccharides, phenolic compounds and other metabolites capable of acting as both reducing and stabilizing agents during NP synthesis. These biomolecules may enhance nanoparticle biocompatibility and antimicrobial performance while avoiding toxic chemical reagents commonly used in conventional synthesis methods. Notably, NPs synthesized using UP extracts have demonstrated very strong antibacterial activity against *Staphylococcus aureus* and *Pseudomonas aeruginosa* [22]. Despite these promising properties, the potential of UP-mediated nanoparticles as antimicrobial agents has so far been primarily demonstrated at the nanoparticle level. Their incorporation into textile substrates, and the extent to which their antimicrobial activity can be retained after immobilization, therefore, remains an important aspect to investigate. In particular, the combined evaluation of the antimicrobial performance and biocompatibility of textiles functionalized with UP-mediated metallic nanoparticles remains limited. This represents a relevant gap in the development of sustainable antimicrobial textile materials for biomedical applications.

In this study, green-synthesized gold (Au@UP) and silver (Ag@UP) nanoparticles produced using UP extracts were immobilized onto cotton and non-woven polyester (PE) gauzes to investigate the influence of nanoparticle composition and textile substrate on antimicrobial performance and biocompatibility. By evaluating two metallic nanoparticle systems on two commonly used textile substrates, we aimed to provide a proof-of-concept for the development of functionalized antimicrobial textiles based on marine-derived nanoparticles. By combining marine-derived nanoparticle synthesis with textile functionalization, this work aims to develop sustainable textile substrates that leverage the potent antimicrobial properties of these nanoparticles, providing an environmentally oriented approach toward the prevention and management of bacterial infections.

## 2. Materials and Methods

### 2.1. Materials

UP seaweed samples were collected from the north-west coast of Spain (42°12′2.9″ N; 8°47′6.2″ W) and stored at −20 °C until use. Polyester-based (PE) (MaiaLab, Faro, Portugal) and cotton gauzes (Disfasan, Barcelona, Spain) were obtained from a local pharmacy. Sodium chloride (NaCl), sodium phosphate dibasic (Na_2_HPO_4_), L-histidine monohydrochloride, lactic acid, sodium hydroxide (NaOH), ethanol, 1,1-difenil-2-picril-hidrazil (DPPH), 6-hydroxy-2,5,7,8-tetramethylchroman-2-carboxylic acid (Trolox), Dulbecco’s modified Eagle’s medium (DMEM), dimethyl sulfoxide (DMSO), 3-(4,5-dimethyl-2-thiazolyl)-2,5-diphenyl-2H-tetrazolium bromide (MTT), chloroauric acid (HAuCl_4_) and silver nitrate (AgNO_3_) were purchased from Sigma-Aldrich (St. Louis, USA). Phosphate-buffered saline (PBS) was prepared using NaCl and potassium chloride (KCl) from Fisher Bioreagents (Waltham, USA) and potassium phosphate monobasic (KH_2_PO_4_) from Panreac AppliChem (Darmstadt, Germany). *Mus musculus* fibroblast cell line L-929 (CCL-1), *Staphylococcus aureus* (ATCC 6538) and *Pseudomonas aeruginosa* (ATCC 10145) were obtained from American Type Culture Collection ((ATCC^®^), Manassas, VA, USA). Luria broth was obtained from Grisp (Porto, Portugal). Fetal bovine serum (FBS) was purchased from Biochrom GmbH (Berlin, Germany).

### 2.2. Methods

#### 2.2.1. UP Extracts Preparation and Nanoparticle Synthesis

The extraction procedure was performed as previously described [22]. Briefly, UP thalli were defrosted, washed, dried, and finely chopped. An aqueous extract (UP extract) was prepared with 1 g_fresh biomass_/mL in endotoxin-free water at 100 °C for 15 min. The biomass was removed by filtration through a 0.22 µm membrane, followed by centrifugation at 4500 rpm for 20 min. The resulting supernatant was subjected to a final filtration step.

Green synthesis of gold (Au@UP) and silver (Ag@UP) nanoparticles was performed as previously reported [18]. Briefly, Au@UP was synthesized at room temperature (RT) under stirring, with chloroauric acid (HAuCl_4_) being slowly added over 24 h. The optimal conditions were achieved using an initial UP extract concentration of 1 g_fresh biomass_/mL and a final HAuCl_4_ concentration of 0.4 mM. Regarding Ag@UP, the synthesis was conducted at 100 °C, with silver nitrate (AgNO_3_) being added under stirring over 30 min. The optimal conditions were obtained using an initial UP extract concentration of 0.5 g_fresh biomass_/mL, with a final solution of 0.25 mM AgNO_3_. Both Au@UP and Ag@UP were frozen at −80 °C and lyophilized in a Christ Beta 2-8 LSC Plus lyophilizer (Martin Christ, Osterode am Harz, Germany) at −64 °C under a vacuum of 0.0060 mbar. A secondary drying step was then applied at −76 °C under a vacuum of 0.0010 mbar to preserve their properties until completely dry, which, in total, took about one day. Before use, the samples were reconstituted with ultrapure water when required, yielding final concentrations of HAuCl_4_ and AgNO_3_ of 0.17 and 0.34 mM.

#### 2.2.2. Characterization of Au@UP and Ag@UP

UV–vis analysis was performed using a BioTek Synergy H1 plate reader (Winooski, USA) at RT in the range of 300–700 nm. Dynamic light scattering (DLS) and electrophoretic light scattering (ELS) analyses were conducted to assess the mean diameter size, polydispersity index and surface charge, expressed as the ζ-potential, of the NPs using a Zetasizer nano ZS (Malvern, UK).

#### 2.2.3. Functionalization of Gauzes with Au@UP and Ag@UP: Exhaustion Method

Lyophilized green-synthesized NPs were reconstituted in ultrapure water to obtain final concentrations of 0.17 or 0.34 mM and sonicated using a 20 kHz probe for 10 s, with a 3 s pulse duration and 35% amplitude, to ensure homogeneity. PE and cotton gauzes of 3 × 3 cm were used for functionalization. The gauzes were immersed in 3 mL of Au@UP or Ag@UP solutions with a non-altered pH and incubated at RT under constant agitation at 100 rpm for 45 min. After incubation, the gauzes were transferred to a Petri Box and cured in a memmert INB 200 oven (Memmert GmbH + Co. KG, Schwabach, Germany) at 180 °C for 7 min. The immersion and curing cycles were repeated five times for the 0.17 mM nanoparticle solutions and three times for the 0.34 mM solutions. Afterwards, the gauzes were washed three times with 3 mL of ultrapure water and air-dried in the same oven at 70 °C until completely dry. Control samples were prepared following the same procedure using UP extract at 0.85 g_fresh biomass_/mL (control for Au@UP) and 1 g_fresh biomass_/mL (control for Ag@UP). Additional control gauzes (cotton and PE) were incubated only with ultrapure water as the functionalizing solution.

**Figure 1:**
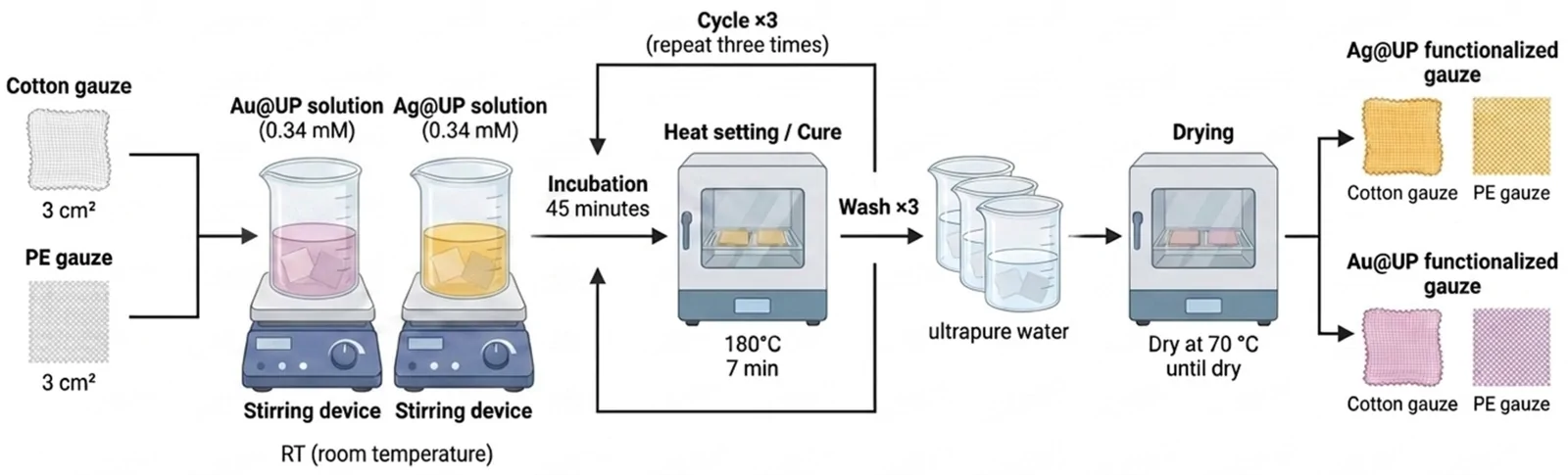
Functionalization by exhaustion employing three subsequent cycles of 45 min of incubation in Au@UP and Ag@UP 0.34 mM solutions at RT and 7 min of heat setting/curing at 180 °C, followed by three washes with ultrapure water and drying at 70 °C. Figure created with FigureLabs Workspace.

The conditions used for the functionalization process were established primarily through a practical optimization based on the visible coloration of the textiles, while avoiding tissue damage at elevated temperatures and incubation times. Regarding the concentrations, as the synthesis of the UP-derived NPs was optimized for a final stock solution of 0.34 mM, this was the highest achievable concentration for this study. The best results were obtained following the method represented in Figure 1.

#### 2.2.4. Functionalization Efficiency

The efficiency of Au@UP and Ag@UP deposition on the textile surfaces was determined by UV–vis analysis using a BioTek Synergy H1 plate reader (BioTek Instruments, Winooski, USA). The nanoparticle concentration in the supernatant was determined at the end of the functionalization procedure. The amount of nanoparticles incorporated into the textile substrates was then calculated by subtracting the concentration remaining in the supernatant from the initial concentration of the functionalization solution. The absorbance measurements were conducted at the previously determined surface plasmon resonance (SPR) wavelengths (530 nm for Au@UP and 430 nm for Ag@UP; Figure S1a,b). The concentration values were then calculated using calibration curves (absorbance versus nanoparticle concentration) for each formulation, within a range of 0 to 0.4 mM and achieving R^2^ values of 0.98 for Au@UP and 0.99 for Ag@UP.

### 2.3. Physicochemical and Structural Characterization of Functionalized Gauzes

#### 2.3.1. Stereo Microscope Imaging

Micrographs of the gauzes functionalized with Au@UP, Ag@UP and UP extracts were obtained using a SZ61/SZ51 Stereo Microscope coupled to an EP50 Wireless LAN Camera (Olympus Corporation, Tokyo, Japan). Images were recorded from both sides of each sample against a white background and from the top view against a black background, using magnifications of 1× and 3×. Control gauzes functionalized only with ultrapure water were used as a control.

#### 2.3.2. Evaluation of Functionalization Degree Through Color Depth

The efficiency of the gauze’s functionalization with Au@UP and Ag@UP was assessed by quantification of color-staining levels (K/S values) at the maximum absorbance wavelength. Measurements were performed using a Colour Reflectance Spectrophotometer (Spectraflash 600 Plus CT, Datacolor International, Lawrenceville, USA) connected to a computer. These measurements were conducted at the maximum wavelength for each sample and were used to evaluate the functionalization efficiency.

#### 2.3.3. Surface Morphology of Functionalized Gauzes: Scanning Electron Microscopy (SEM)

The surface morphology of the functionalized gauzes was examined using a desktop scanning electron microscope (FlexSEM 1000, Hitachi, Japan) to confirm the attachment of Au@UP and Ag@UP onto the gauzes. The samples were mounted on aluminum pin stubs with electrically conductive carbon adhesive tape and sputter-coated with a 2.5 nm gold layer to reduce charging. Imaging was performed under high vacuum at an accelerating voltage of 7, 10 or 15 kV with magnifications of 0.150, 1.5, 2, 4, 4.5, 5 and 7 K×. Measurements of the fiber diameters were conducted using the ImageJ 1.54d software.

#### 2.3.4. Fourier-Transform Infrared Spectroscopy (FTIR-ATR) Analysis

Fourier-transform infrared spectroscopy with attenuated total reflectance (FTIR-ATR) measurements were performed using an ALPHA II Compact FT-IR Spectrometer (Bruker Optics GmbH & Co. KG, Ettlingen, Germany). Samples were placed directly onto the crystal, and spectra were acquired in the range of 4000–500 cm^−1^ with a resolution of 2 cm^−1^. The resulting spectral bands were then analyzed using the OriginPro software (version 10.10) (OriginLab Corporation, Northampton, USA).

#### 2.3.5. Release Profile of NPs from the Functionalized Gauzes

The release profile of Au@UP and Ag@UP from the functionalized gauzes was evaluated using an artificial sweat solution formulated to mimic the human sweat composition [23]. The solution contained 10 g/L of NaCl, 1 g/L of 85% lactic acid, 0.25 g/L of L-histidine monohydrochloride and 1 g/L of sodium phosphate dibasic anhydrous. The pH was adjusted to 4.3 with an alkaline solution of 1 M NaOH.

For this assay, gauzes were cut into 1 × 1 cm pieces and immersed in 5 mL of artificial sweat at 37 °C under constant agitation at 30 rpm. At determined time points, 1, 2, 3, 4, 5, 6, 24, 48 and 72 h, 600 µL of the solution was collected for UV–vis analysis in triplicate. At each time point, the collected volume was reinserted in order to maintain a constant volume and minimize disturbance of the release equilibrium and nanoparticle distribution. Final NP release concentrations were determined using calibration curves generated with a range of 0 to 0.4 mM of each nanoparticle formulation, achieving R^2^ values of 0.98 for Au@UP and 0.99 for Ag@UP.

### 2.4. Bioactive Assessment of Functionalized Gauzes: Antioxidant and Antimicrobial Properties

#### 2.4.1. Radical-Scavenging Activity: DPPH Assay

The DPPH assay was performed as previously described, with slight alterations [24]. Briefly, gauzes were cut into 0.5 × 0.5 cm pieces and placed into tubes. In each tube, 450 µL of 200 µM DPPH was added, followed by incubation in the dark for 2 and 6 h under constant agitation at 200 rpm. At these timepoints, 70 µL of the supernatant was collected, and the absorbance was measured at 515 nm using a BioTek Synergy H1 plate reader (USA). All measurements were performed in triplicate. A positive control was performed with 0.5 mg/mL of Trolox. Both DPPH and Trolox solutions were prepared in 99% ethanol. The percentage of radical-scavenging activity was calculated using the following equation:

$$\%\;DPPH:\frac{{Abs}_{blank}-{Abs}_{sample}}{{Abs}_{blank}}\;\times 100$$

where Abs_sample_ corresponds to the absorbance of the samples, and Abs_blank_ corresponds to the absorbance of wells containing only 200 µM DPPH.

#### 2.4.2. Cytocompatibility of the Au@UP- and Ag@UP-Functionalized Gauzes

*Mus musculus* fibroblast cell line L-929 (ATCC^®^, Manassas, USA) was selected as a reference cell line due to its widespread use in biocompatibility and cytotoxicity studies of biomedical materials, particularly in accordance with ISO 10993 guidelines for the biological evaluation of medical devices [25]. The cells were cultured in high-glucose Dulbecco’s modified Eagle’s medium (DMEM) supplemented with 10% fetal bovine serum (FBS) and 1% antimycotic/antibiotic mix and maintained in a humidified incubator at 37 °C with 5% CO_2_.

The textiles were cut into 1.5 × 1.5 cm pieces and sterilized with UV radiation for 15 min. To obtain conditioned solutions, the functionalized gauzes were immersed in DMEM without fetal bovine serum (FBS) for 24 h at 37 °C. The proportion of medium employed on the gauzes was calculated according to the ISO guidelines for medical devices (ISO 10993-12), at 3 cm^2^/mL [26]. At the same time, the cells were seeded in 96-well plates at a density of 5 × 10^4^ cells/mL and allowed to adhere overnight (100 µL per well). After this period, 10% FBS was added to the conditioned media, which was then transferred to the 96-well plates containing L-929 fibroblasts. After 24 h of incubation at 37 °C and 5% CO_2_, cell metabolic activity was assessed using the MTT assay (3-(4,5-dimethylthiazol-2-yl)-2,5-diphenyltetrazolium bromide) (Sigma, USA).

Formazan crystals were dissolved in 100 µL of an ethanol:DMSO (1:1) solution (DMSO, Sigma, USA), and the absorbance was measured at 570 nm with a microplate reader (Tecan, Infinite® M Plex, Switzerland). The assay included a viability control (fresh culture medium) and a death control (medium containing 30% DMSO).

#### 2.4.3. Antibacterial Activity of the Au@UP- and Ag@UP-Functionalized Gauzes

A direct contact assay was conducted to evaluate the antimicrobial activity of the materials. Samples were tested against *Staphylococcus aureus* (ATCC 6538) (Gram-positive) and *Pseudomonas aeruginosa* (ATCC 10145) (Gram-negative). Bacterial inocula were prepared according to the 0.5 McFarland standard (OD_600_ = 0.08–0.1), corresponding to approximately 1.5 × 10^8^ CFU/mL, following EUCAST/CLSI guidelines. All the assays were conducted in a Bio II Advance Plus laminar flow cabinet (Teslar, Spain), tested according to performance criteria for microbiological safety cabinets (EN 12469:2000) [27].

For the assay, the materials were cut into 1.5 × 1.5 cm squares and sterilized under UV radiation for 30 min. Each sample was placed at the bottom of a well in non-treated, sterile 24-well microplates, and 30 µL of bacterial suspension was added directly onto the material. The microplates were incubated at 37 °C for 30 and 120 min. After incubation, each sample was washed with 970 µL of sterile 1× PBS (8 g of NaCl, 1.44 g of Na_2_HPO_4_, 0.24 g of KH_2_PO_4_, and 0.2 g of KCl, per liter; pH 7.4) and transferred to a 1.5 mL microcentrifuge tube. To determine bacterial survival, 100 µL of the solution was serially diluted at 1:1000 and plated on Luria Broth (LB) agar. The plates were incubated overnight at 37 °C, and colony-forming units (CFUs) were quantified using the ImageJ software (National Institutes of Health, USA) [28].

Samples of cotton and PE gauzes without nanoparticles were used as negative controls.

### 2.5. Statistical Analysis

The data are presented as means ± standard deviation (SD) from three independent experiments. Regarding the fiber diameter in SEM analysis, seven individual fibers were measured for each experimental condition. The significance of the data was determined using the GraphPad Prism^®^ software (version 8.0) by one-way ANOVA with comparisons between each sample and its respective control. The results are expressed as ns *p* > 0.05, * *p* ≤ 0.05, ** *p* ≤ 0.01, *** *p* ≤ 0.001, and **** *p* ≤ 0.0001.

## 3. Results

### 3.1. Characterization of Au@UP and Ag@UP

As reported by us, the physicochemical characterization of Au@UP and Ag@UP nanoparticles was performed using UV–vis spectroscopy, zeta potential analysis, TEM/HRTEM, EDX/EELS, FTIR and size-exclusion chromatography, allowing the evaluation of their optical properties, morphology, crystallinity, surface charge and chemical composition [22]. The nanoparticles were shown to be spherical, crystalline and colloidally stable, with average sizes of 6.8 ± 1.0 nm for Au@UP and 14.1 ± 2.8 nm for Ag@UP, and negative zeta potential values indicating stable dispersions. FTIR and carbohydrate analyses further suggested that polysaccharides and proteins from the UP extract acted as reducing and stabilizing agents during nanoparticle synthesis. To confirm reproducibility and to achieve similar properties regarding Au@UP and Ag@UP, the reconstituted green-synthesized nanoparticles produced with UP extracts were characterized by UV–vis spectroscopy and DLS and ELS analyses. The UV–vis spectroscopy confirmed the characteristic absorbance SPR bands for gold and silver nanoparticles, showing distinct absorbance peaks around 530 nm, for Au@UP, and 430 nm, for Ag@UP. The ζ-potential obtained through ELS revealed highly negative surface charges of −27.7 mV for Au@UP and −34.3 mV for Ag@UP, indicating good colloidal stability. The diameter size obtained by DLS showed mean sizes of 168.2 nm for Au@UP and 180.2 for Ag@UP (Supplementary Data, Figure S1).

### 3.2. Functionalization of Gauzes with Au@UP and Ag@UP: Exhaustion Method

The optimization of the functionalization process of the gauzes was conducted through preliminary practical assays, based on the visual coloration of the functionalized gauzes and on the preservation of textile integrity. The best results were found with 45 min of immersion in either Au@UP or Ag@UP at 0.34 mM, followed by 7 min of curing at 180 °C. This optimization was conducted by varying the incubation (30 to 60 min) and curing times (7 to 10 min), as well as the curing temperature (150 to 180 °C). Different nanoparticle concentrations were also tested (0.17 and 0.34 mM).

#### Functionalization Efficiency

The UV–vis analysis of the supernatant at the end of the functionalization, coupled with the construction of calibration curves for Au@UP and Ag@UP, allowed the determination of incorporated nanoparticle concentrations. Regarding the cotton gauzes, the functionalization with Au@UP and Ag@UP at 0.34 mM achieved ~62% (~0.21 mM) and ~57% (~0.20 mM) of incorporation, respectively. Regarding PE fabrics, 0.34 mM Au@UP and Ag@UP functionalization achieved ~60% (~0.20 mM) and ~56% (~0.19 mM) incorporation, respectively.

### 3.3. Physicochemical and Structural Characterization of Functionalized Gauzes

**Figure 2:**
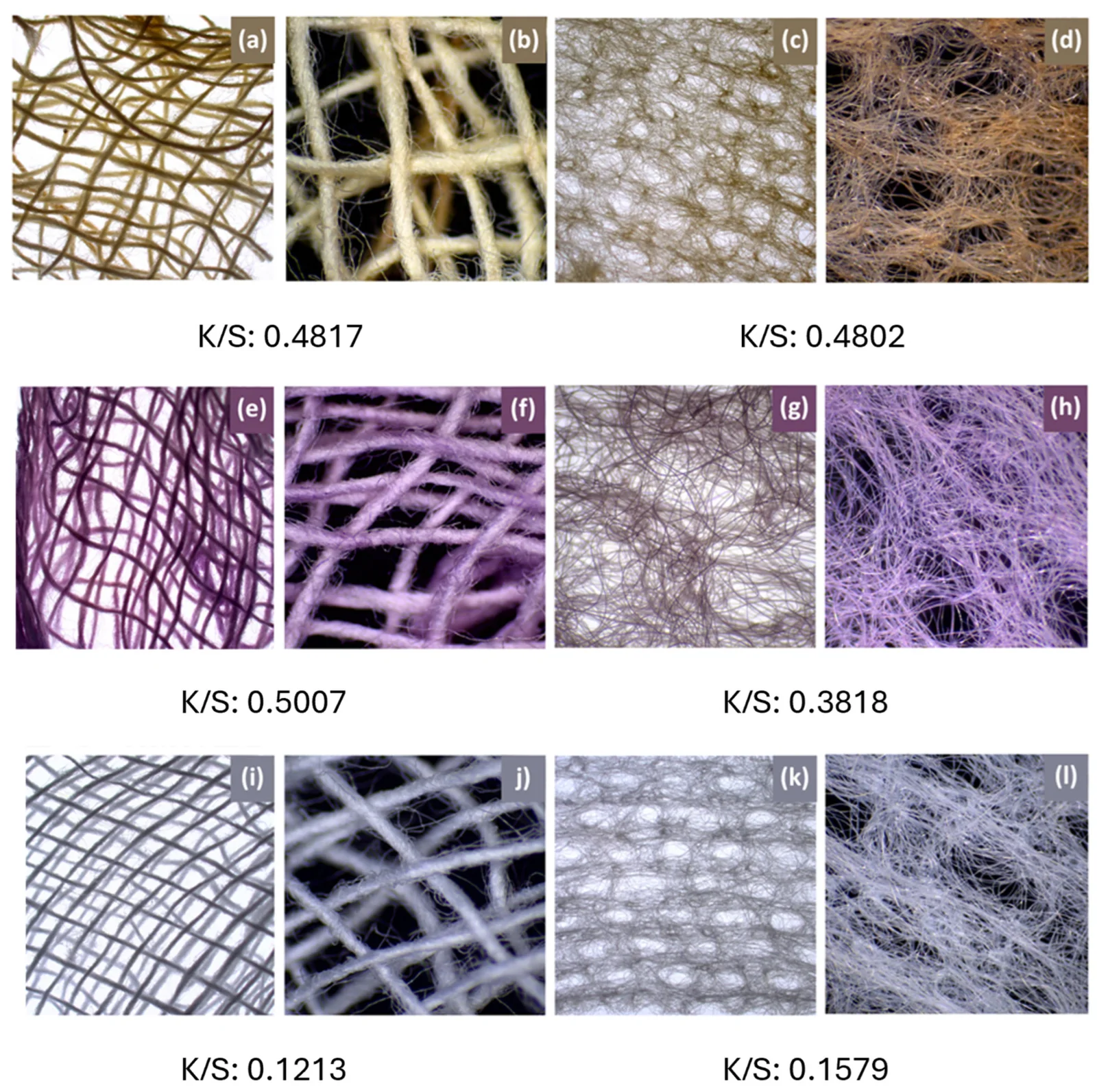
Stereomicroscope images of cotton gauzes (**a**,**b**) and PE gauzes (**c**,**d**) functionalized with Ag@UP 0.34 mM and cotton gauzes (**e**,**f**) and PE gauzes (**g**,**h**) functionalized with Au@UP 0.34 mM. Cotton (**i**,**j**) and PE (**k**,**l**) gauzes treated with ultrapure water, as control. Magnifications of 1× (first and third columns) and 3× with a black background (second and fourth columns) used for imaging. The respective K/S values are presented under images of each condition.

The coloration of the functionalized gauzes was examined using a stereo microscope, allowing a more detailed observation of the differences between each condition. The resulting images clearly show the distinct colors instilled by each nanoparticle type, as the gauzes acquired the characteristic color of the corresponding functionalizing solution: lilac for Au@UP and yellow for Ag@UP. To quantify this parameter, the color strength was analyzed before and after functionalization, using the K/S parameter. The clear differences observed between the functionalized samples and the control gauzes are supported by the higher K/S values, which reflect the intensity and depth of the colored materials by correlating light absorption with light-scattering coefficients. The functionalization efficiency appeared comparable for both fabrics treated with Ag@UP (~0.48). However, a substantially higher value was obtained for Au@UP-functionalized cotton (0.5007) compared with Au@UP-functionalized PE (0.3818). Images of the functionalized gauzes and their corresponding K/S values are shown in Figure 2.

**Figure 3:**
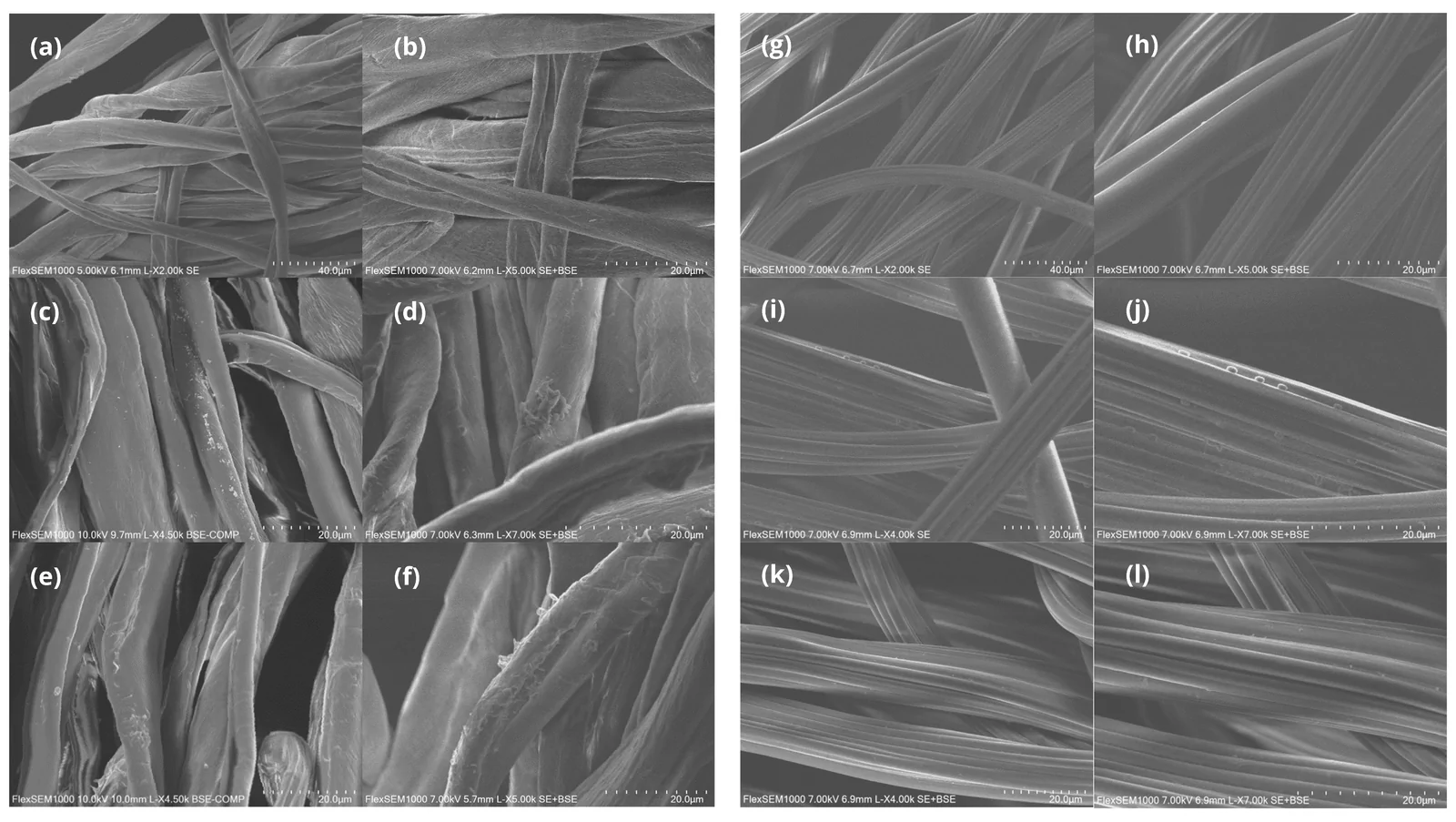
Scanning electron microscopy (SEM) images at different magnifications ranging from 0.15 to 7 K of the gauzes before and after functionalization. Cotton gauzes functionalized with ultrapure water as control (**a**,**b**), 0.34 mM Au@UP (**c**,**d**) and 0.34 mM Ag@UP (**e**,**f**). PE gauzes functionalized with ultrapure water as control (**g**,**h**), 0.34 mM Au@UP (**i**,**j**) and 0.34 mM Ag@UP (**k**,**l**).

The surface morphology of the samples was examined by scanning electron microscopy (SEM) (Figure 3). The resulting images demonstrated that both Au@UP and Ag@UP were successfully coated onto the cotton and PE gauzes, as evidenced by increased surface roughness due to the deposition of NPs and UP extract compounds. This phenomenon was more pronounced in the cotton gauzes. Some aggregates were also observed. However, since particle size strongly influences adhesion, larger agglomerates are expected to be more easily leached from the textile surface [29]. Measurements of cotton fibers demonstrated increases of 1.06 µm (Ag@UP, *) and 2.84 µm (Au@UP, ****) when compared with the untreated cotton control. For PE gauzes, more slight changes were observed, with increases of 1.05 µm (Ag@UP, ns) and 1.48 µm (Au@UP, *). These differences reinforce the successful deposition of Au@UP, Ag@UP and the extract compounds on the surfaces of the fabrics.

**Figure 4:**
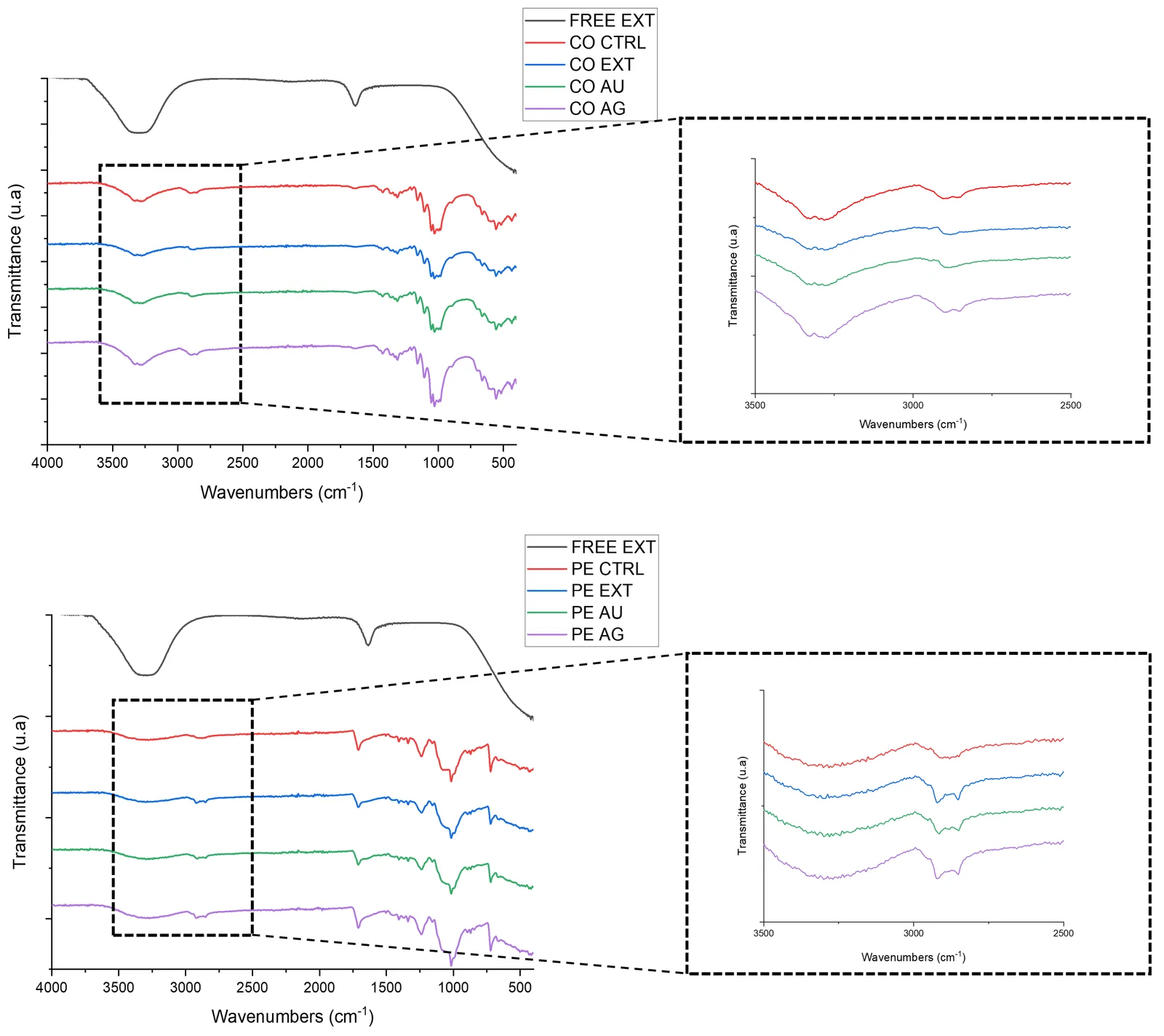
Fourier-transform infrared spectroscopy–attenuated total reflectance (FTIR-ATR) analysis of cotton (**up**) and PE (**down**) gauzes functionalized with UP extract at 1 g_fresh biomass_/mL and Au@UP and Ag@UP at 0.34 mM. Non-treated gauzes and free UP extract were used as controls.

FTIR-ATR analysis was performed to characterize the functionalized textile samples and assess changes in chemical interactions. The analysis detected the presence of chemical groups typical of UP extracts, responsible for stabilizing and capping the metal nanoparticle core, as well as functional groups present in the textiles [30]. The FTIR spectra of the free UP extract, functionalized gauzes and their respective textile controls are presented in Figure 4. FTIR analysis revealed that the functionalized gauzes exhibited similar spectral profiles when compared with their respective fabric controls, indicating that the textile matrices were preserved upon treatment. In all samples, the characteristic bands of the textile remained dominant, particularly the broad O–H stretching region around 3300 cm^−1^ and the C–H stretching vibrations at 2900 cm^−1^ [31]. Although slight variations were observed in the fingerprint region (1200–900 cm^−1^), no distinct absorption bands or peaks can be attributed to the UP extract, according to its spectrum. This behavior was observed for both textiles, as the spectral characteristics of the fabrics masked any potential signals from the deposited extract, Au@UP or Ag@UP. However, some slight differences can be noticed between functionalized textiles, suggesting changes in the chemical composition of the textile substrates due to possible interactions with the functionalizing agents.

### 3.4. Release Profile of NPs from the Functionalized Gauzes

**Figure 5:**
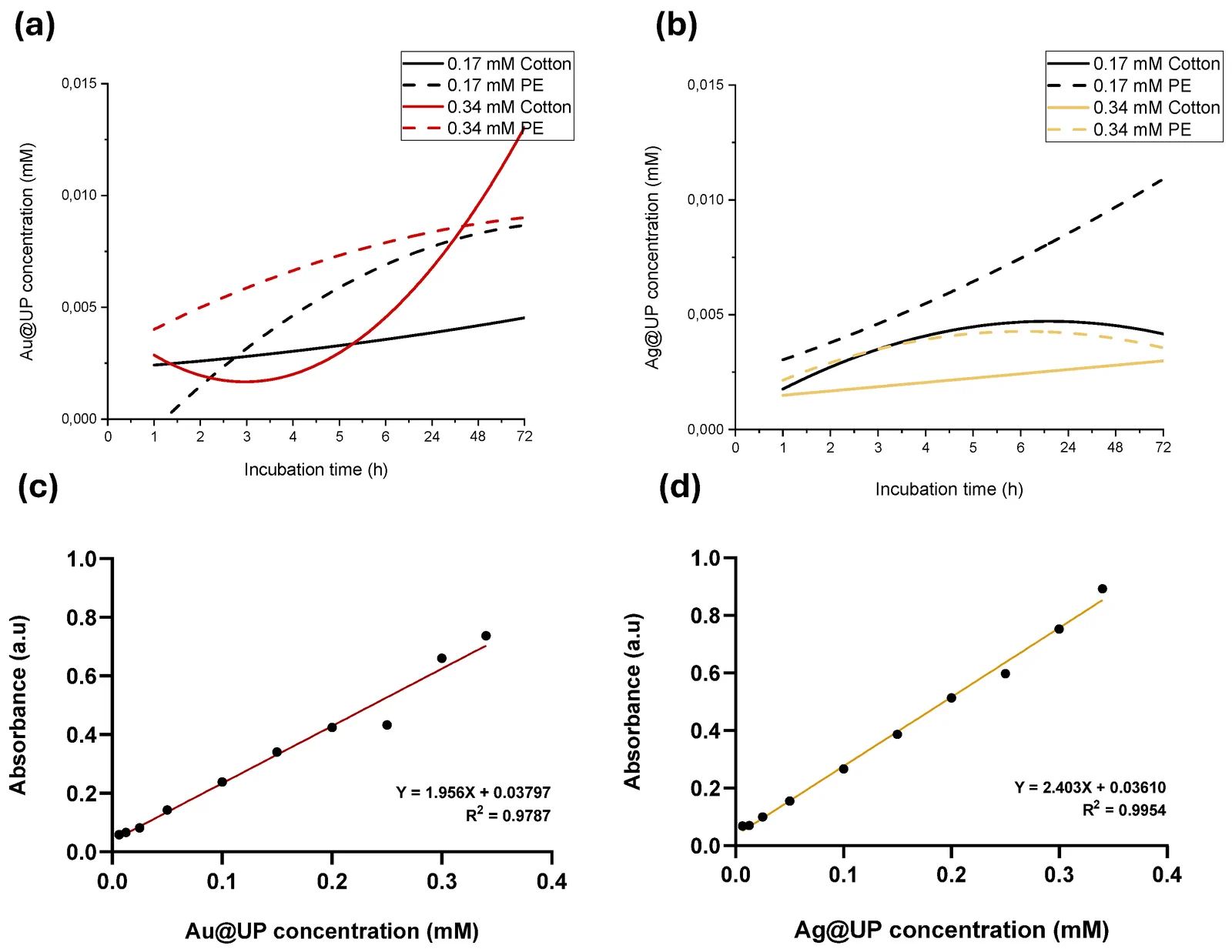
Cumulative release concentration measured using artificial sweat on functionalized cotton and PE gauzes with Au@UP (**a**) and Ag@UP (**b**). Values were obtained from calibration curves prepared with Au@UP (**c**) and Ag@UP (**d**) solutions and are represented as polynomial trend lines.

A cumulative release assay was carried out to obtain insight into the leaching behavior of the NPs from the gauzes under conditions simulating human skin contact. For this purpose, an artificial sweat solution with an adjusted pH was used. The absorbance of the supernatant was measured at predefined time points, and a calibration curve was established to determine the concentration of NPs in the supernatant at each interval. The results are presented in Figure 5. For Ag@UP, UV–vis endpoint measurements revealed that samples functionalized with the lower NP concentration (0.17 mM) exhibited a more pronounced and faster release, particularly at later time points. This behavior suggests that the number of functionalization cycles—five for 0.17 mM and three for 0.34 mM—may influence the strength of the NPs’ attachment to the textile surface. In contrast, this trend was not observed for the Au@UP-functionalized gauzes, where the higher concentration (0.34 mM) displayed a greater release for both textile types. The release profiles of both Au@UP and Ag@UP displayed fluctuations throughout the assay. The seemingly inconsistent variations in the NP concentration in the supernatant and absorbance over time may indicate that the NPs undergo cyclic attachment and detachment processes. However, similar fluctuations were also observed in the control gauzes, suggesting that this behavior may also be related to the leaching of intrinsic textile compounds rather than to nanoparticle release alone.

### 3.5. Bioactive Assessment of Functionalized Gauzes: Antioxidant and Antimicrobial Properties

**Figure 6:**
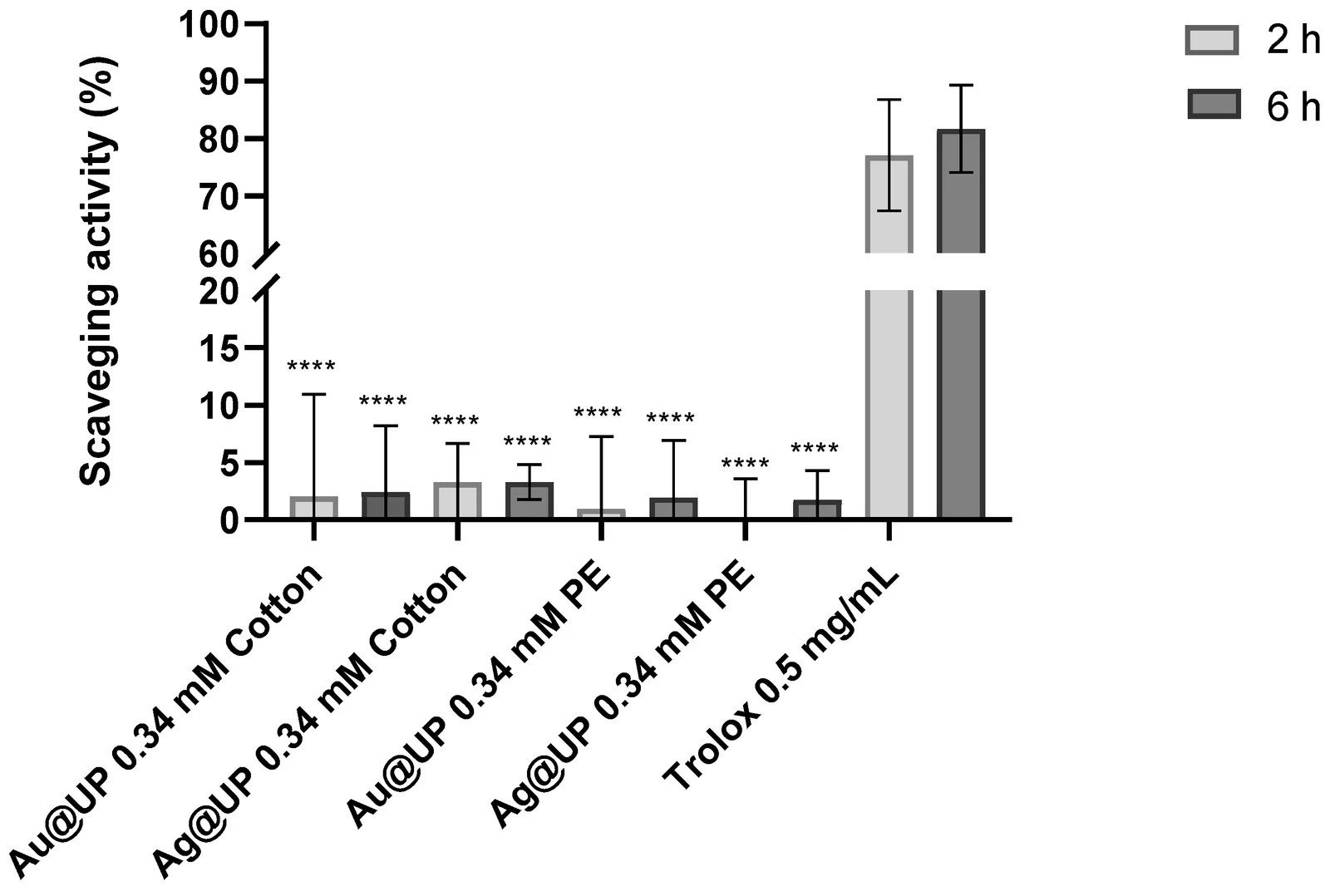
DPPH radical-scavenging activity (%) of the functionalized gauzes and their respective controls. The assay was conducted under agitation (200 rpm) for 2 and 6 h of incubation. Values are presented after subtraction of the corresponding textile control and are represented as the means from three independent experiments (*n* = 3). Trolox at 0.5 mg/mL was used as the positive control. Statistical differences were compared with the positive control at the respective time point under every condition (**** for *p* < 0.0001). No statistical differences between functionalized gauzes were observed. Error bars represent the standard deviation.

To assess the antioxidant potential of the functionalized gauzes, a DPPH radical-scavenging assay was conducted. Since the antioxidant activity of both the UP extracts and the UP-mediated nanoparticles had already been reported [22], the objective here was to determine whether the attachment to the textiles would alter this activity. Given the minimal release observed during the initial hours of the release study, the DPPH assay was performed under agitation, and supernatants were collected after 2 and 6 h of incubation. The scavenging activity of the control was subtracted from all conditions to obtain clearer results (Figure 6). After 2 h of exposure to the functionalized gauzes, all the tested conditions exhibited higher scavenging activity than the untreated textiles, with the exception of the 0.34 mM Ag@UP PE sample. Nonetheless, all samples showed increased activity at the endpoint measurement (6 h), with cotton gauzes tending to exhibit slightly higher activity than the PE ones, although no statistical differences were identified between conditions. Additionally, all samples demonstrated a significantly lower antioxidant activity when compared with the positive control performed with 0.5 mg/mL of Trolox (*p* < 0.0001).

**Figure 7:**
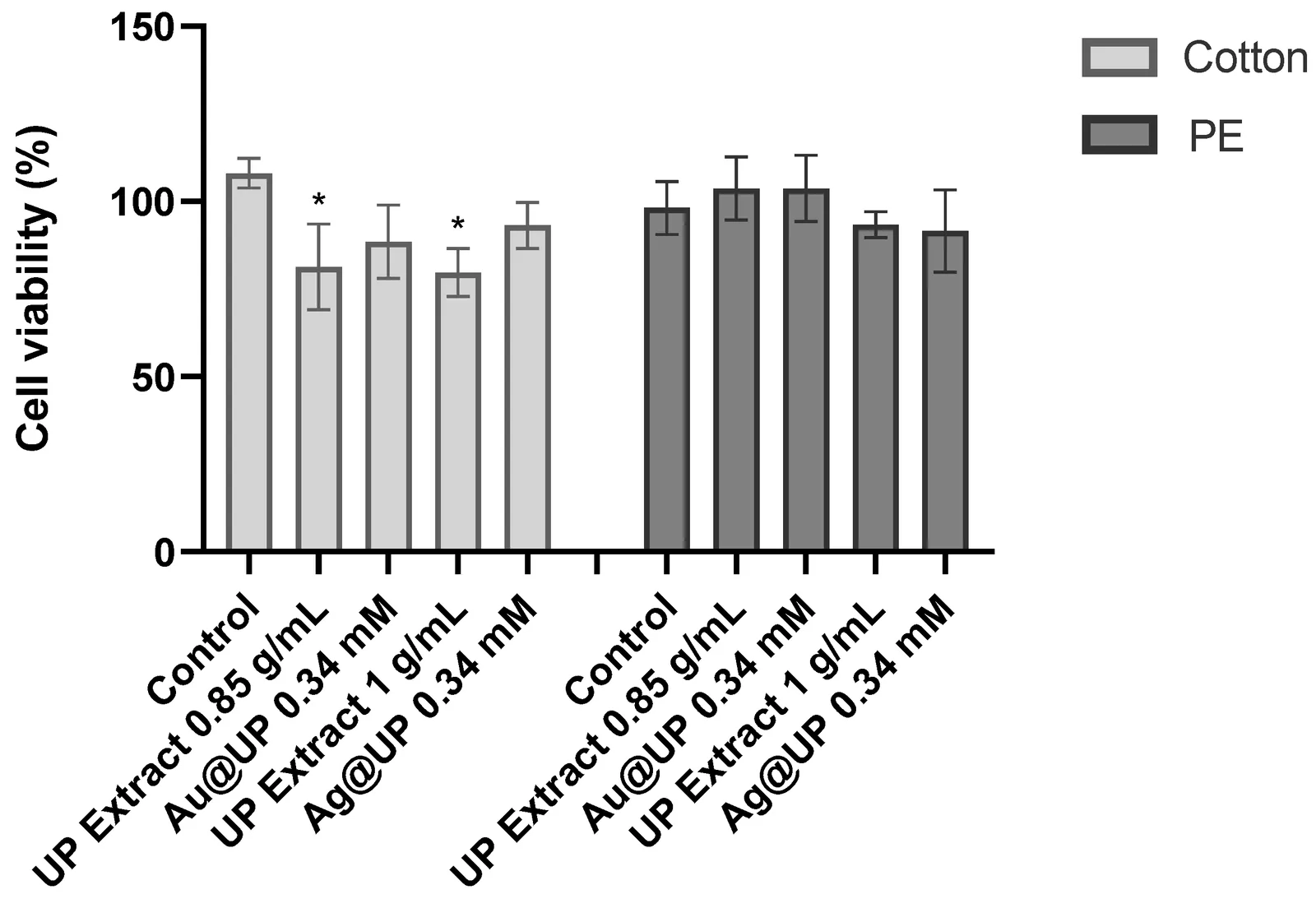
Cell viability (%) after 24 h of incubation in culture medium pre-conditioned with functionalized gauzes. Viability was assessed using the MTT assay and normalized to the live control values cultured in fresh medium (100%). Extract control gauzes were functionalized with UP extracts at 0.85 and 1 g_fresh biomass_/mL for Au@UP and Ag@UP, respectively. * *p* < 0.05 compared with the respective control. No further statistically significant differences were observed between any conditions tested. Error bars represent standard deviation.

As part of evaluating the potential of these functionalized textiles for biomedical applications, an indirect-contact cytotoxicity assay was performed using the widely used reference, murine fibroblast cell line L-929. For this purpose, the culture medium was preconditioned by incubation with the samples for 24 h prior to cell exposure. After incubation, metabolic cell activity was assessed using the MTT assay, and the results were normalized to the life control (100% viability in fresh culture medium). Additionally, gauzes functionalized with crude UP extract (0.85 g_fresh biomass_/mL for Au@UP and 1 g_fresh biomass_/mL for Ag@UP) were included as controls to evaluate whether the extracts themselves exhibited cytotoxicity. The results are presented in Figure 7. IC50 values were not emphasized because it was not possible to achieve 50% inhibition within the tested concentration range of NPs, as a constraining factor of the nanoparticle synthesis process.

For the functionalized cotton gauzes, a significant decrease in cell viability was observed for both crude UP extract conditions (0.85 and 1 g_fresh biomass/mL_) (*p* < 0.05), compared with the untreated gauzes. Meanwhile, none of the functionalized gauzes with Au@UP or Ag@UP displayed cytotoxicity. This suggests that the UP extracts display greater cytotoxicity than the nanoparticles synthesized with them, although no significant differences were detected between the NP-treated samples and their respective UP extract controls (0.85 g_fresh biomass_/mL for Au@UP; 1 g_fresh biomass_/mL for Ag@UP). For the PE-functionalized gauzes, no significant differences were observed among any of the tested conditions or controls. Likewise, no statistically significant differences were identified between the two textiles.

To evaluate the antimicrobial activity of the functionalized gauzes, a direct-contact assay was performed by applying Staphylococcus aureus and Pseudomonas aeruginosa suspensions directly onto the samples. These bacteria were used as models of Gram-positive and Gram-negative bacteria, respectively. An initial optimization assay was conducted by varying the incubation time (30 or 120 min) and NP concentration (0.17 or 0.34 mM). The highest antimicrobial activity was achieved with Ag@UP-functionalized gauzes, for both textile substrates and against both bacterial strains (Supplementary Data, Tables S1 and S2). The optimal conditions corresponded to the highest NP concentration (0.34 mM) and the most prolonged incubation time (120 min) (Supplementary Data, Table S3).

**Figure 8:**
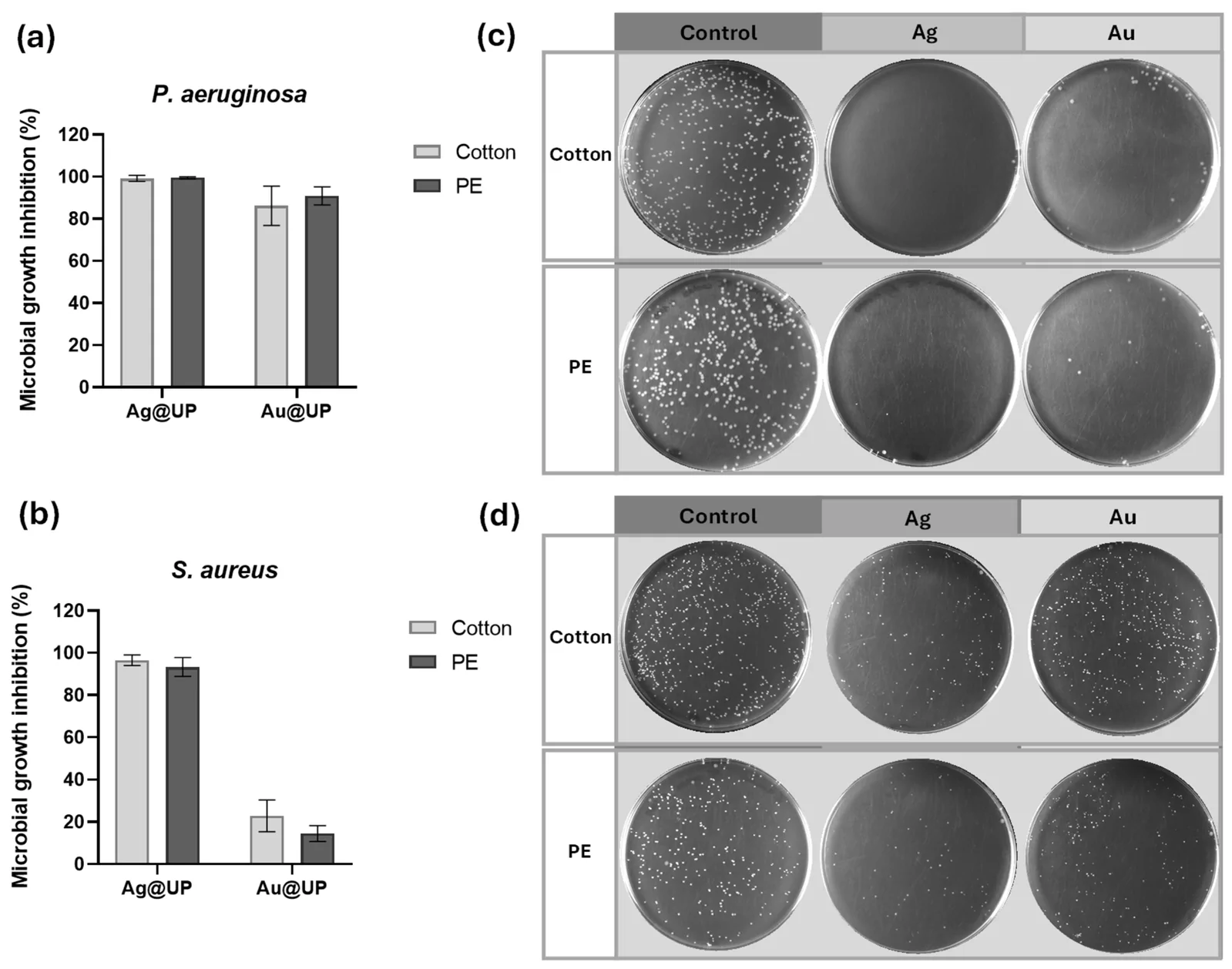
Antimicrobial activity of cotton and PE gauzes functionalized with Au@UP and Ag@UP (0.34 mM) after 120 min of contact. Microbial growth inhibition (%) against *Pseudomonas aeruginosa* (**a**) and *Staphylococcus aureus* (**b**) was determined by CFU counting and calculated relative to untreated cotton and PE gauzes. Representative agar plate images after exposure to functionalized gauzes are shown for *P. aeruginosa* (**c**) and *S. aureus* (**d**). Error bars represent standard deviation.

Antimicrobial testing (Figure 8) demonstrated that Ag@UP-functionalized gauzes exhibited high antimicrobial efficacy, achieving inhibition values exceeding 90% against both S. aureus and P. aeruginosa. Au@UP also showed strong antimicrobial activity against P. aeruginosa (~90%), whereas substantially lower inhibition was observed against S. aureus (~15% for PE and ~20% for cotton). These results suggest that the differences in bacterial cell wall composition may influence the efficacy of the functionalized materials.

## 4. Discussion

Nanotechnology-based antibacterial strategies have received considerable attention in recent years due to their unique modes of action and lower likelihood of developing resistance [9]. As textiles typically provide an ideal environment for microorganism growth, with adequate moisture and oxygen availability, the need to develop multi-functional textiles is emerging in response to increasing awareness of health and hygiene [3,32]. Therefore, in this study, silver and gold green-synthesized NPs were used to functionalize widely used medical textiles [6,7]. The synthesis of Ag@UP and Au@UP was carried out employing UP aqueous extracts in order to obtain nanoparticles with antioxidant and antibacterial activities previously reported in [22]. Further characterization was performed, confirming the reproducibility of the synthesis. The functionalization was performed on cotton and non-woven PE gauzes using the exhaustion method.

The exhaustion method is affected by several parameters, such as incubation time and temperature, the type and concentration of the functionalization solution, and post-treatment conditions, such as heat-setting/curing temperature [33]. Incubation times usually range from 5 to 90 min. Temperature, considered the most decisive factor, is reported to enhance the process efficacy [34]. To maintain the functionalization process as environmentally friendly and sustainable as possible, incubation was performed at room temperature. Given the high thermal stability of metal nanoparticles, the heat setting/curing of the gauzes was carried out at a higher temperature than commonly reported for this method [35,36,37]. The curing process is crucial due to the rearrangement of dipole interactions and hydrogen bonds, which may help to stabilize and attach the NPs onto the textile surface [38,39]. Accordingly, the gauzes were successfully functionalized through exhaustion, using cycles of 45 min of incubation in the respective NP solution, followed by curing for 7 min at 180 °C. These were the conditions that exhibited stronger final coloration of the gauzes. Morphological alterations and shrinkage of the gauzes were monitored throughout the process to ensure textile damage was avoided.

The efficacy of the functionalization was initially confirmed by determining the incorporation of Au@UP and Ag@UP through UV–vis analysis of the supernatant and through visual and stereomicroscopy inspection. Textile color changes, from pale yellow to lilac for Au@UP or yellow/orange for Ag@UP, were readily observed during the gauze functionalization process. These colors are associated with the SPR, because in metallic nanoparticles, surface electrons generate an intense absorption band in the UV–vis spectrum when exposed to light [40,41]. As such, the UV–vis analysis was conducted mainly on these parts of the NP formulation’s spectra. The lilac and yellow also correspond to the coloration of the original Au@UP and Ag@UP formulations. As such, their appearance indicates that the particles were successfully attached to the textiles [42,43].

To further confirm the NP-derived adsorption onto the gauzes, the color strength values (K/S) were obtained through the absorption coefficient (K) and the scattering coefficient (S), demonstrating the depth of color on functionalized fabrics. The results demonstrate nanoparticle deposition through a significant increase in K/S values for Au@UP- and Ag@UP-functionalized textiles, while much lower color depth values were obtained for the cotton and PE controls. This has been previously reported for silver-nanoparticle-functionalized cotton fabrics [44]. Since Au@UP and Ag@UP absorb light at the previously described wavelengths due to the SPR phenomenon, the reflectometer can be used as an indirect measure of nanoparticle adhesion through absorbance increase [45]. The difference in values between textiles on Au@UP samples might be attributed to polyester-based textiles usually presenting fewer free active groups as well as higher hydrophobicity when compared with cotton, promoting less nanoparticle attachment [46]. However, this behavior was not noticeable on Ag@UP-functionalized textiles, probably because these nanoparticles present a lower surface charge when compared with Au@UP, exhibiting higher colloidal stability and, therefore, lower reactivity [22,47]. Additionally, the presence of UP extract compounds bound to the different textile substrates might also be a key factor for the differences found in nanoparticle adhesion efficiency [32]. Nevertheless, the results indicated higher color adsorption on cotton gauzes, particularly those functionalized with Au@UP.

SEM analysis was conducted primarily to evaluate significant changes in the textiles’ morphology, with the original smooth surface of the untreated textiles becoming rough upon functionalization. The deposition of UP extract compounds, consistent with an increase in fiber diameter upon functionalization, was confirmed by SEM, especially on functionalized cotton gauzes. Under some conditions, nearly spherical structures could be observed, which suggested the successful deposition of the metallic NPs. However, further characterization would be required to infer the nature of these structures. These alterations in surface morphology have been described previously regarding nanoparticle-functionalized textiles such as cotton and polyester [44,48]. These results suggest the successful deposition of Au@UP and Ag@UP, as well as UP extract compounds, on the textile surfaces.

Since the metallic NPs cannot be detected by FTIR due to the lack of molecular vibrations under infrared radiation, the main spectral differences observed in the functionalized gauzes are attributed to the UP extract components [49]. Although slight alterations seem to appear in the analyzed spectra, FTIR analysis of the functionalized gauzes demonstrated high dominance by the textile matrix. This masking phenomenon has been previously reported on natural extract-functionalized textiles, aligning with our results on the fabric matrix groups dominating FTIR spectra [50,51]. Nevertheless, in both tested fabrics, slight changes can be observed in the O-H absorption band region, suggesting that these groups may be responsible for effectively distributing and stabilizing the interactions between the NPs or the UP extract compounds and the textile substrates [32,52]. The successful deposition of the UP extract compounds, present on the nanoparticle formulations, is also supported by the increased presence of organic groups such as -CH_2_ and -CH_3_, associated with the presence of carboxylic acids, lipids and hydrocarbon chains in the functionalized samples [53,54]. This effect was more pronounced in the Ag@UP-functionalized gauzes, possibly because silver nanoparticle solutions contained a higher extract concentration (1 g_fresh biomass_/mL) than the gold counterpart (0.85 g_fresh biomass_/mL), owing to the dilution adjustments required to achieve an equivalent concentration of 0.34 mM of nanoparticles. These results align with those of previous reports, as these groups are widely present on proteins that play a crucial role in stabilizing and capping agents during green synthesis [32,54].

The physical bonding between textiles and NPs is believed to be caused by electrostatic interactions, which are weak interactions that might allow NPs’ detachment under certain environmental triggers [32,55]. This property is very interesting in this study, as the antimicrobial potential of Au@UP and Ag@UP might require their detachment from textiles. The cumulative release assay was evaluated by UV–vis spectroscopic analysis, measuring the absorbance of the supernatant at different time points over a 72 h incubation period. An artificial sweat solution was used to simulate skin-contact conditions and to assess the leaching behavior of the nanoparticles from the functionalized gauzes during direct contact with human skin [56]. The functionalized gauzes with different Au@UP and Ag@UP concentrations demonstrated a fluctuating release profile, suggesting that both the number of cycles involved in the functionalization—five for 0.17 mM and three for 0.34 mM—and the type of NPs influence their attachment to the textiles. This observation aligns with the understanding that surface functionalization represents a critical parameter governing particle migration [57]. Moreover, higher nanoparticle concentrations are associated with increased UP extract contents, resulting in a greater amount of compounds rich in hydroxyl groups that promote stronger and more extensive interactions with the textile surface [32,55,58]. Our results did not fit the usual controlled release models, probably due to the absorbance fluctuations; so, a simple line-fitting model was used to evaluate release upon textile control interference subtraction. These fluctuations may be explained by the leaching of impurities from the textiles [59], as similar profiles were detected on the control gauzes, and similar behaviors were previously reported, even when employing comparable artificial sweat solutions [23]. Nevertheless, it is possible to notice a slow-release tendency of the textiles functionalized with either Au@UP or Ag@UP, with only residual concentrations of NPs being leached over time. This phenomenon may prolong the desired bioactivities, such as antibacterial activity, while reducing the risk of toxicity associated with gold and silver NP exposure, especially at higher concentrations. The release assay reinforces the hypothesis that the NPs might behave through dynamic processes, not following a typical release profile.

**Figure 9:**
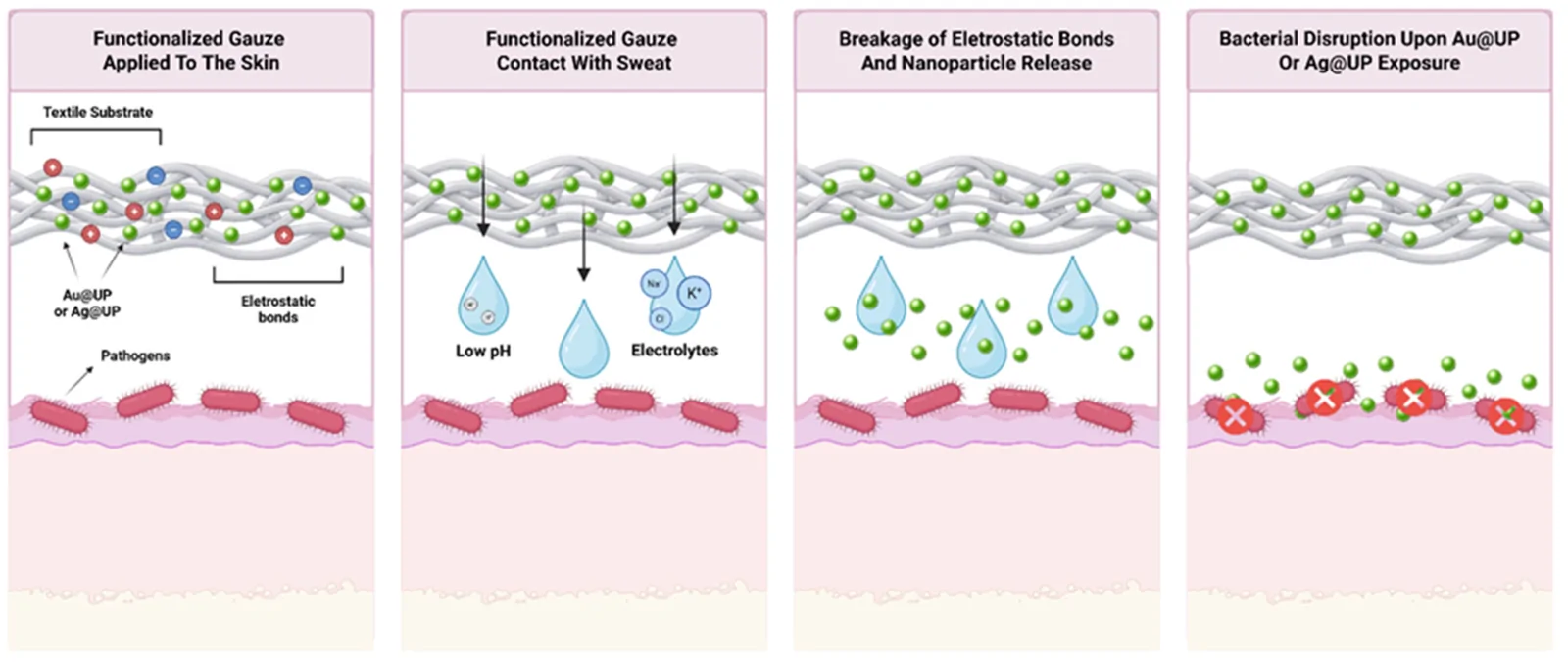
Suggested mechanism of release profile and antimicrobial action of Au@UP- and Ag@UP-functionalized textiles when in contact with the skin. Figure created with BioRender.

Following these results, we hypothesize that the NPs interact with the textile substrates through electrostatic forces, allowing their dynamic attachment and detachment during both the functionalization cycles and the release assay. This behavior has been previously reported by many authors, who suggest that the binding of metallic NPs occurs through van der Waals interactions [32,51]. These interactions, when in contact with sweat—including exposure to parameters such as low pH and the presence of electrolytes—may be weakened, allowing the release of the NPs and their action as antimicrobial agents against pathogens. This pattern has also been previously described for silver-NP-functionalized textiles, as acidic environments promoted NPs’ leaching mostly in the form of silver ions [57,58]. The presence of free ions may also be a key factor in weakening electrostatic interactions by increasing ionic strength. In these conditions, electrolytes may compete with bound species for interaction sites, reducing the strength and stability of electrostatic associations [59]. These authors further suggest that nanoparticles’ release profile is directly proportional to their initial concentration during functionalization. Our suggested mechanism is, therefore, presented in the following Figure 9.

Regarding the antioxidant testing of the functionalized gauzes, neither the UP extracts nor the NPs exhibited significant antioxidant potential. This finding is consistent with previous reports showing that the antioxidant capacity of aqueous UP extracts is relatively low compared with other brown seaweeds and is further reduced by 2.5- and 1.5-fold for Au@UP and Ag@UP, respectively, as antioxidant compounds in the extract play a crucial role during NP synthesis [18,60]. This decrease in antioxidant activity may be attributed to the loss of hydroxyl groups during the synthesis of metallic nanoparticles [61]. Nevertheless, we suggest that the masking of the bioactives’ antioxidant activity might also be due to a combination of factors, such as partial degradation during the thermal-curing treatment (180 °C) and low mobility upon immobilization on the textile’s matrices [61,62]. As the scavenging capacity is related to the arrangement and availability of hydroxyl groups, the attachment of NPs to the textile substrates could further hinder their antioxidant potential [63]. This might also be associated with potential textile–oxygen ligations, which could mask the bioactives’ action and enhance nanoparticle retention on the textile fibers [64]. This is consistent with the DPPH assay results obtained for the functionalized gauzes, which showed a peak at around 3% for cotton gauzes functionalized with Au@UP at 0.34 mM.

Since the aim of this work was to assess the suitability of the functionalized gauzes for biomedical applications without posing toxicity risks, the murine fibroblast cell line L-929 was exposed to culture media preconditioned with the samples. The results showed that the cotton textiles functionalized with crude UP extracts induced some degree of cytotoxicity after overnight exposure. In contrast, this effect was not observed for cotton textiles functionalized with either Au@UP or Ag@UP. This reduced toxicity of NPs compared with their synthesis extracts in fibroblast-like cells has been previously reported for both gold and silver particles [65,66]. Moreover, cotton textiles functionalized with different NPs, including green-synthesized silver NPs obtained using natural extracts, have been shown to be safe for biological applications [67,68]. A marked reduction in cell viability was observed for cotton gauzes compared with PE gauzes. This behavior may be explained by the attachment of metabolites originating from the UP extract to the cotton substrate, resulting in higher concentrations than on the PE substrate.

The strong antimicrobial activity of Ag@UP-functionalized textiles against *S. aureus* (Gram-positive) and the even greater activity observed against *P. aeruginosa* (Gram-negative) are in agreement with previous reports on silver and other green-synthesized metallic particles, which typically show higher resistance in Gram-positive bacteria [69,70,71]. This difference in susceptibility is usually attributed to variations in the bacterial cell wall structure, as the thinner peptidoglycan layer on Gram-negative bacteria facilitates penetration of metallic ions into the cell [72,73]. A similar behavior was observed for Au@UP-functionalized gauzes, which exhibited a strong decrease in antimicrobial activity against *S. aureus*, compared with *P. aeruginosa.* In the context of green synthesis, we hypothesize that the observed antimicrobial activity may result from a synergistic effect between the metallic core of the nanoparticles and the phytochemical composition of the UP extract. This hypothesis is consistent with previous reports on these NPs in a free state, as the minimum inhibitory concentration against these bacteria was 7.88 µg/mL for Ag@UP and 11.81 µg/mL for Au@UP, whereas for the crude UP extract, antimicrobial activity was not detected [18].

The differences in antimicrobial activity observed for Au@UP and Ag@UP nanoparticles may be due to distinct modes of action. Silver nanoparticles exhibit intrinsic antimicrobial effects through direct interactions with metabolic systems involved in the respiratory chain or through interference with DNA synthesis [74]. Additionally, silver ions are reported to induce oxidative stress [12]. These effects are largely mediated by the release of positively charged silver ions, which interact with the bacterial cell membrane, inducing the formation of silver pits [74]. Gold particles, conversely, generally display weaker antimicrobial effects, as their activity is mainly derived from the presence of antimicrobial conjugates, such as bioactive natural compounds, rather than from the metallic core itself [74,75,76]. It is important to note that the different cell wall architectures of the tested bacteria and the nanoparticle’s modes of action are unlikely to solely explain the discrepancy found in antimicrobial activity, as it could also be explained by other factors, such as the physicochemical properties of each nanosystem.

The incorporation of green-synthesized NPs into textiles to impart antimicrobial properties has been extensively explored. Silver NPs synthesized using plant extracts, such as *Acorus calamus* and microalgae extracts, such as *Spirulina*, as well as gold NPs produced from aqueous plant extracts of *Coleus aromaticus*, *Croton sparsiflorus* and *Ginkgo biloba Linn*, have demonstrated effective microbial growth inhibition when incorporated into cotton and polyester-based fabrics [32,43,73,77,78]. However, information regarding the use of brown seaweed extracts for the green synthesis of NPs and textile functionalization remains limited. Cotton fabrics functionalized with zinc oxide nanoparticles from aqueous extracts of the brown seaweed *Padina* sp. exhibited only moderate antimicrobial activity [79]. Moreover, these materials required approximately 5 h of incubation to achieve significant inhibition of *P. aeruginosa*, in contrast with the UP-mediated nanoparticles used in this study, which achieved approximately 90% inhibition within just 2 h. Furthermore, preliminary assays with Ag@UP nanoparticles demonstrated that considerable antimicrobial activity was attained after only 30 min of incubation. Our previous work on UP-mediated silver and gold NPs demonstrated strong antimicrobial properties, which were retained upon incorporation into both cotton and PE gauzes [18].

Although silver salts remain the most reported precursors to synthesize nanoparticles acting as antimicrobial agents when applied to textile substrates, many other metallic cores have been explored to obtain these materials. Cuprous oxide nanoparticles synthesized using *Myrciaria dubia* juice and incorporated into textiles showed complete (100%) antibacterial activity against both *S. aureus* and *E. coli* [80]. Cotton fabrics functionalized with copper, magnesium, and nickel oxide nanoparticles synthesized using *Cynomorium coccineum* extract reduced *S. aureus* growth by 45%, 85%, and 91%, respectively, after only 2 h of incubation [10]. Accordingly, studies have shown that cotton- and polyester-based fabrics functionalized with biosynthesized zinc oxide and silver NPs retain antimicrobial activity, whereas untreated textiles exhibited no bioactivity either before or after washing [29,81]. Several other metals have also been described as promising agents against microbial growth. However, direct comparisons are challenging due to different metrics and experimental conditions. Compared with these systems, our findings highlight the strong antibacterial potential of Au@UP- and, particularly, Ag@UP-functionalized fabrics. These results are especially noteworthy considering the consistent activity of Ag@UP against both Gram-positive and Gram-negative bacterial models, demonstrating its considerable potential as an effective antimicrobial textile functionalization.

## 5. Conclusions

Green-synthesized silver and gold NPs were previously obtained using aqueous extracts of the invasive seaweed UP and shown to exhibit strong antimicrobial activity. The present study extended this work by demonstrating the functionalization of widely used medical textiles, namely, cotton- and polyester-based gauzes, with these biogenic NPs. The textiles acquired the characteristic coloration of the functionalizing solutions, indicating NP attachment by electrostatic interactions and stabilization by hydroxyl groups present in UP extract compounds. This attachment was confirmed through UV–vis spectroscopy, K/S measurements, FTIR spectroscopy and SEM analysis, which revealed the presence of extract-derived functional groups and efficient nanoparticle deposition on the textile substrates. The functionalized gauzes exhibited residual release, with a dynamic attachment–detachment mechanism that may contribute to the prolonged durability of their bioactive properties, particularly antimicrobial activity. Moreover, no cytotoxic effects were observed in fibroblasts following exposure to the functionalized gauzes. Regarding the functionalized gauzes, Ag@UP demonstrated remarkable inhibitory effects against both *S. aureus* and *P. aeruginosa*, while Au@UP showed strong activity exclusively against P. aeruginosa within just 2 h of contact. Overall, these results highlight UP-mediated NP-functionalized textiles as a sustainable and promising approach for inhibiting microbial growth and preventing bacterial infections.

## Data Availability

All data can be made available upon request to the corresponding authors through the institutional resource https://datarepositorium.uminho.pt/. Raw data regarding the DLS and ELS analyses are available at https://doi.org/10.34622/datarepositorium/26GZLS (accessed on 10 August 2026).

## Supplementary Materials

The following supporting information can be downloaded at https://www.mdpi.com/article/10.3390/nano16171112/s1. Figure S1: UV–vis spectroscopy of Au@UP (a) and Ag@UP (b) nanoparticles. ζ-potential (c); mean diameter and polydispersity index (d) determined by ELS and DLS for both nanoparticles. Table S1: Optimization of the antimicrobial assay conducted with different incubation times (30 or 120 min) and NP concentrations (0.17 or 0.34 mM) using Pseudomonas aeruginosa as a Gram-negative bacterial model. Untreated gauzes were used as a control. Table S2: Optimization of the antimicrobial assay conducted with different incubation times (30 or 120 min) and NP concentrations (0.17 or 0.34 mM) using Staphylococcus aureus as a Gram-positive bacterial model. Untreated gauzes were used as a control. Table S3: CFU count from the optimization of the antimicrobial assays performed with the functionalized gauzes with varying incubation times (30 or 120 min) and NP concentrations (0.17 or 0.34 mM) and using *Staphylococcus aureus* (Gram-positive) and *Pseudomonas aeruginosa* (Gram-negative) as bacterial models. Untreated gauzes were used as a control. NC stands for non-countable.

## Abbreviations

The following abbreviations are used in this manuscript:

ANOVA	Analysis of variance
ATCC	American Type Culture Collection
CFUs	Colony-forming units
CLSI	Clinical and Laboratory Standards Institute
DLS	Dynamic light scattering
DMEM	Dulbecco’s modified Eagle’s medium
DMSO	Dimethyl sulfoxide
DPPH	2,2-diphenyl-1-picrylhydrazyl
EDX	Energy-dispersive X-ray spectroscopy
EELS	Electron energy-loss spectroscopy
ELS	Electrophoretic light scattering
EUCAST	European Committee on Antimicrobial Susceptibility Testing
FBS	Fetal bovine serum
FTIR	Fourier-transform infrared spectroscopy
FTIR-ATR	Fourier-transform infrared spectroscopy–attenuated total reflectance
HRTEM	High-resolution transmission electron microscopy
IC_50_	Half-maximal inhibitory concentration
K/S	Colour strength parameter
LB	Luria broth
MTT	3-(4,5-dimethyl-2-thiazolyl)-2,5-diphenyl-2H-tetrazolium bromide
NPs	Nanoparticles
OD_600_	Optical density at 600 nm
PBS	Phosphate-buffered saline
PE	Polyester
RT	Room temperature
SD	Standard deviation
SEM	Scanning electron microscopy
SPR	Surface plasmon resonance
TEM	Transmission electron microscopy
UP	*Undaria pinnatifida*
UV–vis	Ultraviolet–visible spectroscopy
