# Supplementary material for: Sustainable Antimicrobial Textiles Functionalized with Gold and Silver Nanoparticles Biosynthesized Using Undaria pinnatifida Extracts
Source: Nanomaterials (Basel). 2026 Sep 3;16(17):1112. doi: 10.3390/nano16171112 (PMC13567326; doi:10.3390/nano16171112)
Supplement: Supplementary file 1 [file nanomaterials-16-01112-s001.zip › nanomaterials-4525195-supplementary.pdf]

Supporting information

Sustainable antimicrobial textiles functionalized with gold and silver nanoparticles biosynthesized using *Undaria pinnatifida* extracts

João Abreu[a,b,c,d], Mário Fernandes[a,b], Ana Rita Bragança[a,b], Bruna Silva[a,b], Diana Rocha[c,d], Raúl Machado[a,b], Artur Ribeiro[c,d], Maria Carmen Rodríguez-Argüelles[e], Carla Silva[c,d*], Andreia Gomes[a,b*]

[a] Centre of Molecular and Environmental Biology (CBMA)/Aquatic Research Network (ARNET) Associate Laboratory, Universidade do Minho, Campus de Gualtar, 4710-057 Braga, Portugal;

[b] Institute of Science and Innovation for Sustainability (IB-S), Universidade do Minho, Campus de Gualtar, 4710-057 Braga, Portugal

[c] Centre of Biological Engineering, University of Minho, Campus de Gualtar, 4710-057 Braga, Portugal

[d] LABBELS—Associate Laboratory, 4710-057 Braga, 4800-058 Guimarães, Portugal

[e] Departamento de Química Inorgánica, Universidade de Vigo, 36310 Vigo, Spain

*Authors to whom correspondence should be addressed.

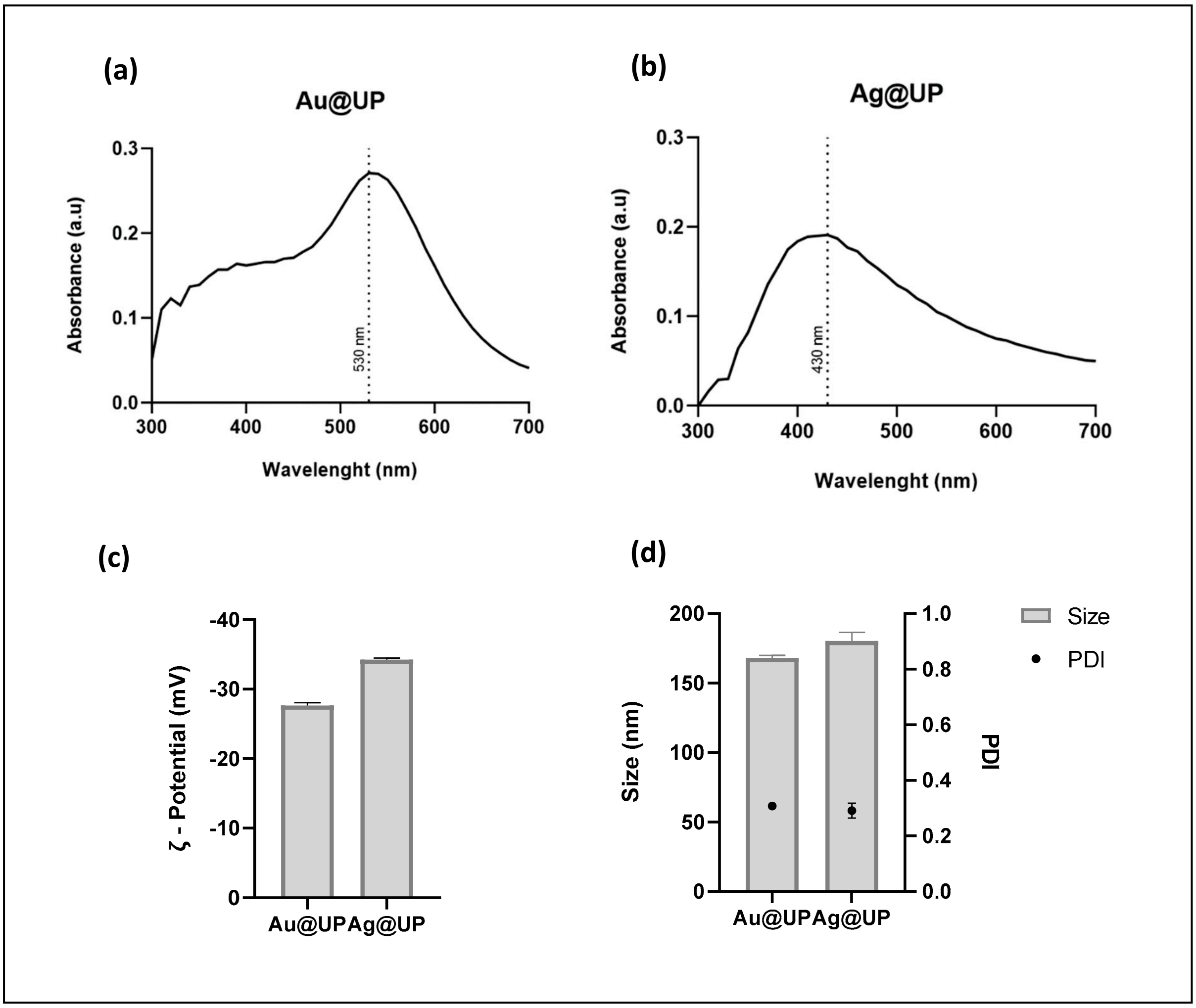

Figure S1. UV–vis spectroscopy of Au@UP (a) and Ag@UP (b) nanoparticles. ζ-potential (c); mean diameter and polydispersity index (d) determined by ELS and DLS for both nanoparticles.

Table S1. Optimization of the antimicrobial assay conducted with different incubation times (30 or 120 min) and NP concentrations (0.17 or 0.34 mM) using *Pseudomonas aeruginosa* as a Gram-negative bacterial model. Untreated gauzes were used as a control.

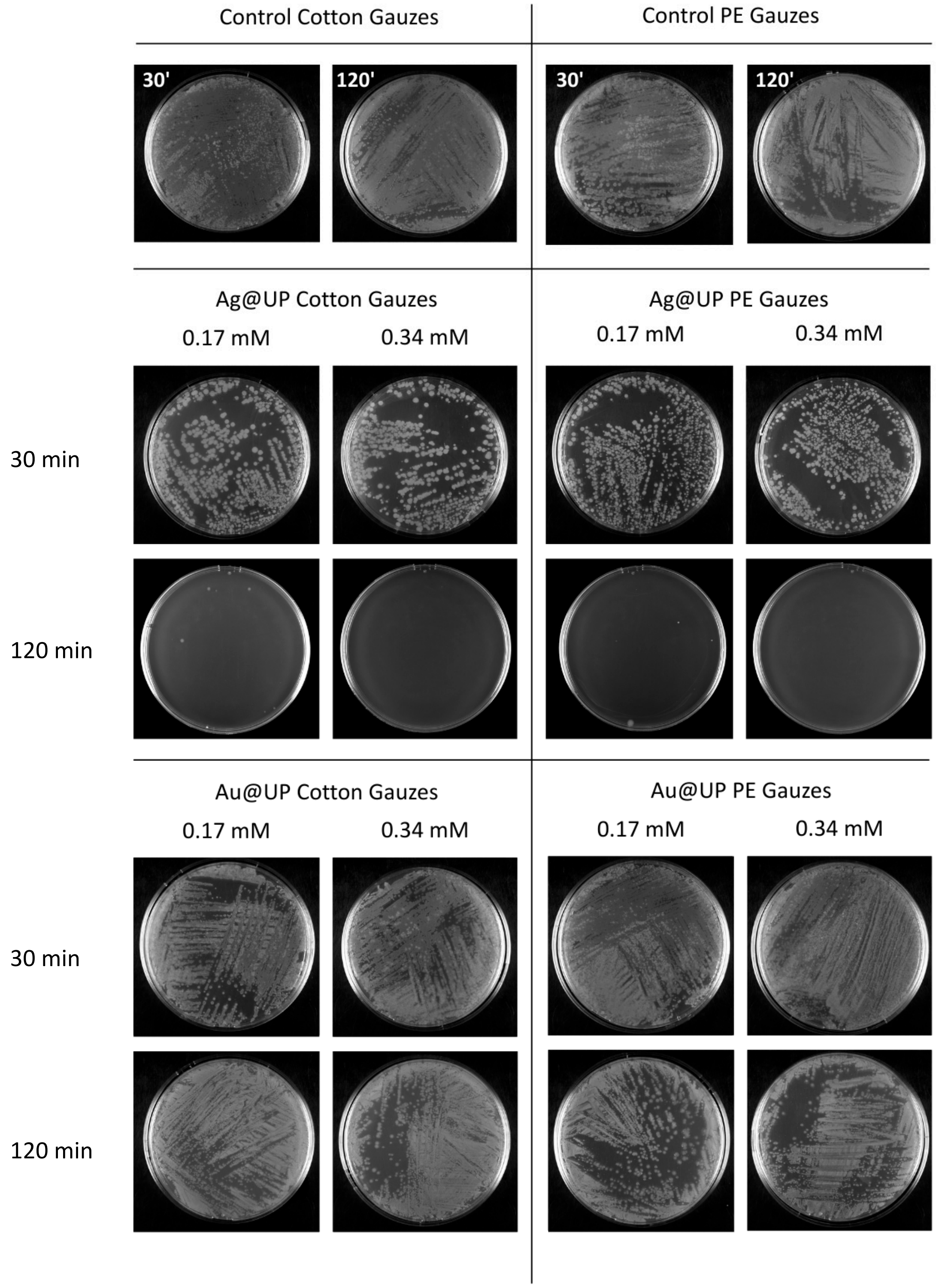

Table S2. Optimization of the antimicrobial assay conducted with different incubation times (30 or 120 min) and NP concentrations (0.17 or 0.34 mM) using *Staphylococcus aureus* as a Gram-positive bacterial model. Untreated gauzes were used as a control.

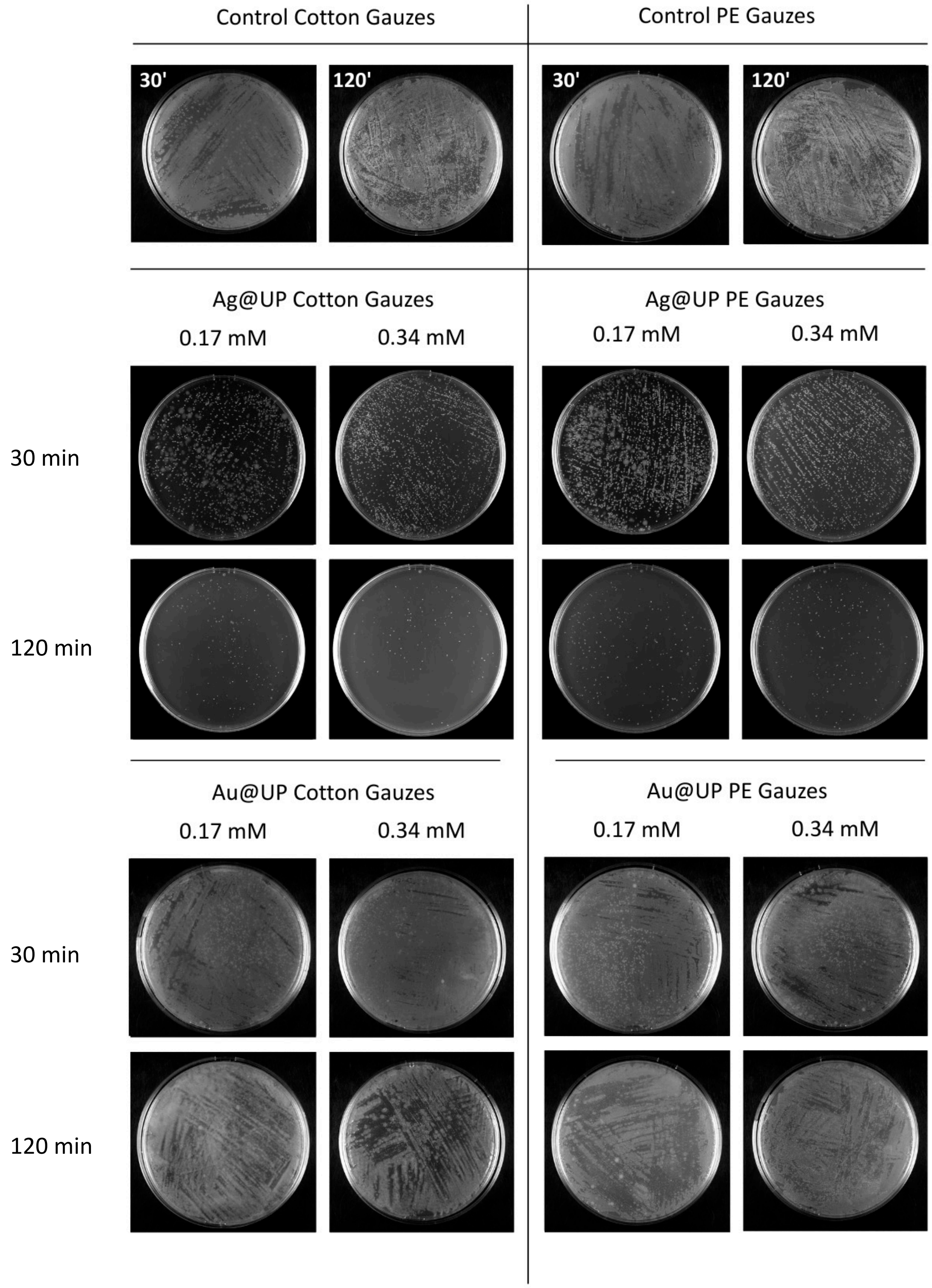

Table S3. CFU count from the optimization of the antimicrobial assays performed with the functionalized gauzes with varying incubation times (30 or 120 min) and NP concentrations (0.17 or 0.34 mM) and using *Staphylococcus aureus* (Gram-positive) and *Pseudomonas aeruginosa* (Gram-negative) as bacterial models. Untreated gauzes were used as a control. NC stands for non-countable.

			P. aeruginosa **(Gram -)**		***S. aureus*** **(Gram +)**	
**Gauze Material**	**NP Type**	**[NP] mM**	**Incubation Time**		**Incubation Time**	
			**30 min**	**120 min**	**30 min**	**120 min**
**Cotton**	**-**	-	NC	NC	NC	NC
	**Au@UP**	0.17	NC	NC	NC	NC
		0.34	NC	NC	NC	NC
	**Ag@UP**	0.17	NC	7	NC	203
		0.34	NC	0	NC	131
**PE**	**-**	-	NC	NC	NC	NC
	**Au@UP**	0.17	NC	NC	NC	NC
		0.34	NC	NC	NC	NC
	**Ag@UP**	0.17	NC	5	NC	308
		0.34	NC	0	NC	290
